# Micronutrient in hyperphenylalaninemia

**DOI:** 10.1016/j.dib.2015.07.026

**Published:** 2015-08-01

**Authors:** Vanesa Crujeiras, Luís Aldámiz-Echevarría, Jaume Dalmau, Isidro Vitoria, Fernando Andrade, Iria Roca, Rosaura Leis, Ana Fermandez-Marmiesse, María L. Couce

**Affiliations:** a Unit of Gastroenterology and Nutrition, Department of Pediatrics, Hospital Clinico Universitario de Santiago, Travesía da Choupana s/n, 15706 Santiago de Compostela, A Coruña, Spain; b Unit of Metabolism, Department of Pediatrics, Hospital de Cruces, Group of Metabolism, Biocruces Health Research Institute, CIBERER, Plaza de Cruces s/n, 48903 Barakaldo, Vizcaya, Spain; c Unit of Metabolopathies, Hospital Universitario la Fe, Bulevarsur s/n, 46021 Valencia, Spain; d Unit of Diagnosis and Treatment of Congenital Metabolic Diseases, S. Neonatology, Department of Pediatrics, Hospital Clinico Universitario de Santiago, Travesía da Choupana s/n, 15706 Santiago de Compostela, A Coruña, Spain; e Unit of Diagnosis and Treatment of Congenital Metabolic Diseases, S. Neonatology, Department of Pediatrics, Hospital Clinico Universitario de Santiago, CIBERER, Health Research Institute of Santiago de Compostela (IDIS), Travesía da Choupana s/n, 15706 Santiago de Compostela, A Coruña, Spain

## Abstract

The data presented here are the biochemical parameters of 156 patients with hyperphenylalaninemia. PKU patients, who, in order to maintain optimal serum Phe concentrations, receive dietary treatment consisting of a diet low in natural protein supplemented with special low protein foods and a Phe-free amino acid mixture, vitamins and minerals. The obtained data reflects a high percentage of patients with prealbumin and selenium deficiencies, as well as an increased level of folic acid. This data article is related to the research article entitled, “Vitamin and mineral status in patients with hyperphenylalaninemia”, by Crujeiras et al. [1].

**Table:** 

Specifications Table	
Subject area	*Metabolic diseases*
More specific subject area	*Vitamins and minerals in phenylketonuria*
Type of data	*Table*
How data was acquired	*By clinical history*
Data format	*Analyzed data*
Experimental factors	*None applied*
Experimental features	-Tandem mass-spectrometry and standard procedures of laboratory
Data source location	PKU patients from three Spanish Centers: Hospital Universitario La Fe-Valencia, Hospital Universitario de Cruces-Basque Country and Hospital Clínico Universitario de Santiago-Galicia.
Data accessibility	Data is supplied in this article

**Table:** 

Value of the data	
•	A high percentage of selenium deficits as well as a high percentage of folic acid overloads are detected in PKU patients treated, which should lead us to reflect on whether to adjust international standards supplements formulated milks.

## 1 Data, experimental design, materials and methods

The population comprised PKU patients from three Spanish Centers: Hospital Universitario La Fe-Valencia, Hospital Universitario de Cruces-Basque Country and Hospital Clínico Universitario de Santiago-Galicia. Patients were followed in their center from diagnosis to date. We included both patients diagnosed by newborn screening as well as those diagnosed later. Those who underwent sporadic monitoring were excluded, as well as those cases in which informed consent was not signed by parents and/or patients.

The variables collected for each patient were: age, sex, phenotype (mild hyper-Phe (MHPA) 120–360 μmol/L; mild-moderate PKU 360–1200 μmol/L (MPKU); classic PKU>1200 μmol/L (CPKU) (according to US Guidelines [2], disease detection (early and late diagnosis), annual median blood Phe levels (target blood Phe levels considered adequate for children under 6 years of age were <360 µmol/L; <480 µmol/L between 6 and 10 years and ≤600 µmol/L in older patients), anthropometric measurements (weight, height and BMI), phenylalanine tolerance and BH4 treatment in the BH4 responsive patients; blood chemistry (total protein, prealbumin, electrolytes, selenium (Se), zinc (Zn), B_12_, folic acid, ferritine, 25-hydroxy vitamin D (25-OHD).

Micronutrients levels evaluated present the following reference intervals (valid for any patient age): Total protein: 6.3–8.5 g/dL; calcium (Ca): 8.7–10.8 mg/dL; phosphorus (P): 2,6-5.9 mg/dL, prealbumin: 21–41 mg/dL; vitamin B_12_: 180–1900 pg/mL; folic acid: 3–17.5 ng/mL; 25-OHD: 20–42 ng/mL; ferritin: 14–325 ng/mL; Zn: 65–140 µg/dL; Se: 60–125 µg/L

## 2 Data

In PKU patients, micronutrient deficiencies but no clinical symptoms have been reported, mainly for Zn, Se, Fe, folate and B_12_ [3–5]. In this study 127 patients had altered values in one or several markers evaluated (Table 1), 58.33% were from CPKU, 21.67% from MPKU and 20% from MHPA patients. Interestingly, from these patients with altered values, the 84.16% had adequate adherence to diet.

Total protein, Ca, P, B_12_, ferritin, and Zn levels were in the normal range in almost all patients. Nevertheless, Palb was decreased in 34.6% of patients (74% from PKU) being the 94.4% of them below 18 years old and having the 96.3% an adequate adherence to treatment. Se was found to be diminished in 25% of patients, 76.9% of them with classic PKU but only in 2.56% of patients under BH4 treatment.

An adequate intake of Ca, P and 25-OHD is necessary for optimal bone accretion but it is not enough for bone normal development because several studies have showed that protein intake deficiency can influence the bone mass [6,7]. Our patients generally had adequate levels of Ca and P, but there were found low levels of 25-OHD in 14% of them. The 25-OHD was found altered in 14% of total patients. No deficiency of folic acid was found in our cohort, but was found to be above the upper limit in 39% of patients, 65.6% with classic PKU and 82% of them with adequate adherence to diet.

Patients treated with BH4 presented lower levels of B_12_ with respect to non-treated (536 vs 694 pg/mL, p: 0.0094). No other anthropometric parameters were found to be correlated with any variable in our study.

We noted in our study a high percentage of PKU patients with good adherence to diet with Prealbumin deficiency and total protein normal. Also a high percentage of PKU patients with Se deficiency had folic acid increase. On the contrary, only el 2.56% de patients under BH4 treatment showed Se deficiency.

## Figures and Tables

**Table 1:** Characteristics of Hyperphenylalaninaemia patients with values of the biochemical parameters studied.

P	Sex	Age	BMI	Diag	Tre. D/T	TD	Phe tol mg/d	Phe median µM	Prot g/dL	Prealb mg/dL	Ca mg/dL	P mg/dL	Fer ng/mL	B12 pg/mL	Folic ng/mL	25-OHD ng/mL	Zn µg/dL	Se µg/dL
1	F	2y 11m	N	CPKU	D	E	345	138	6.6	18↓	9.9	5.6	135	853	24↑	40	99	47↓
2	M	19y 3m	↑	CPKU	D	L	217	380	7.9	25	9.8	3.3	36	823	24↑	38.7	117	74
3	M	2y 7m	N	CPKU	D	E	223	258	7	17↓	10	5.6	29	789	15.1	40.2	76.5	106
4	F	2y 7m	↑↑	CPKU	D	E	218	123	6.4	14↓	10.2	5.6	30	444	13	26	56.6↓	38.6↓
5	M	18y 2m	N	CPKU	D	E	469	408	7.5	30	10.1	3.9	37	723	24↑	37.4	78.5	32.3↓
6	M	28y 9m	↑	CPKU	D	E	367	1032	7.5	36	10.2	4	32	272	8.6	25.5	119	52.7↓
7	F	1y 2m	↑	CPKU	D	E	118	162	6.5	14↓	9.2	4	84	696	18.7↑	36.2	81.5	68.7
8	F	15y 2m	N	CPKU	D	E	454	480	7.7	28	10.4	4	126	405	17.3	19.1↓	70	70
9	F	2y 4m	↓	CPKU	D	E	243	90	6.7	16↓	10.1	5.2	28	934	18.5↑	40	78.5	62.5
10	F	13y 1m	↑↑	CPKU	D	E	390	474	6.8	22	9.6	5.2	5↓	347	24↑	34.4	82.5	69.5
11	F	3y 3m	N	CPKU	D	E	289	225	7	14↓	10.3	4.7	40	835	13.2	29.3	59.7↓	19.6↓
12	M	4y 10m	N	CPKU	D	E	379	207	6.8	17↓	10	5.1	28	462	17.2	27.8	106	62
13	F	92y 7m	↑	CPKU	D	E	440	522	6.6	34	9.3	3.9	40	980	20↑	41.3	145↑	69.5
14	F	9y 6m	N	CPKU	D	E	153	300	7.5	19↓	10.1	4.9	52	617	16.9	26.4	71.5	57↓
15	M	7y	N	CPKU	D/T	E	1162	336	6.7	21	10.4	4.5	34	779	18.6↑	48↑	76.5	68.7
16	M	2y 6m	↓	CPKU	D	E	473	348	6.4	22	9.8	5	21	468	24↑	40.6	76.5	58.7↓
17	F	15y 6m	N	CPKU	D	E	447	450	7.7	19↓	10	4.6	32	388	5.68	27.2	101	71
18	M	39y	↑	CPKU	D	E	205	777	6.5	31	9.4	2.3↓	89	354	16.5	27.6	113	51.7↓
19	M	5y 8m	↑↑	CPKU	D	E	242	330	6.9	30	9.3	4.5	40	761	12	24	80	36.1↓
20	M	3y 2m	↑↑	CPKU	D	E	194	204	6.8	21	10.1	9.9↑	24	560	–	34.2	78.5	–
21	F	14y 9m	↑	CPKU	D	E	432	366	6.9	18↓	10.1	3.8	48	421	16.7	28.7	65	57↓
22	M	2y 9m	N	CPKU	D	E	448	243	6.6	20↓	10.1	4.8	32	656	20.2↑	31.9	78.5	34↓
23	M	18y 10m	N	CPKU	D	L	503	252	7.6	26	9.9	4.8	9.9↓	824	16.9	36.9	103	68.7
24	F	23y 11m	↑	CPKU	D	E	320	381	7.4	28	9.3	3.5	61	628	16.3	41.3	144↑	68.7
25	M	3y 9m	↑	CPKU	D	E	381	444	6.1↓	19↓	9.8	5.9	97	838	24↑	32.2	125	68.7
26	F	34y 9m	↑↑	CPKU	D	E	409	1158	7.7	21	9.6	3	39	524	6.4	30.4	120	40.18↓
27	M	5y 10m	↑	CPKU	D	E	425	258	7.2	16↓	9.6	5	13↓	675	15.1	19↓	73.5	74.9
28	F	30y 4m	N	CPKU	D	E	771	972	6.4	21	8.5↓	2.7	22	301	11.5	20.2	75.5	–
29	M	3y 7m	N	CPKU	D	E	226	192	6.3	16↓	10.7	5.9	8↓	679	16.3	36.5	80.5	68.7
30	M	23y	↑	CPKU	D	E	256	846	7.2	37	10.1	2.3↓	91	471	14.4	41.9	145↑	77.2
31	M	9m	N	CPKU	D	E	250	90	6.4	20↓	10.3	6.4↑	267	1201	17.2	37.5	170↑	77.2
32	F	17y 4m	↑↑	CPKU	D	L	169	576	6.8	22	9.8	3.3	23	937	24↑	20	75.5	49.4↓
33	M	10y 6m	N	CPKU	D	E	55	156	6.4	17↓	9.8	3.3	127	423	16	31.8	93	71
34	F	6y 4m	N	CPKU	D	E	537	156	7.3	20↓	10.4	5.5	42	600	14.3	42	71.5	135↑
35	M	5y 11m	↑	CPKU	D	E	403	138	6.5	18↓	9.9	5.1	24	372	16.9	52↑	74.5	124
36	M	5y 1m	N	CPKU	D	E	489	384	6.7	19↓	10	4.5	27	1875	18.5↑	43.2↑	77	54.8↓
37	M	42y 6m	↑	CPKU	D	L	121	888	7.6	24	9.2	–	73	660	30↑	26	69.2	60
38	F	5y 5m	↑↑	MPKU	D/T	E	1069	198	6.7	18.4↓	10	–	35	501	30↑	33	87.3	42↓
39	F	4y 1m	N	MPKU	D	E	438	117	7	22.8	10.2	–	34	766	28↑	36	67.2	48↓
40	M	26y 4m	↑	MPKU	D	E	430	300	7	19.1↓	9	–	108	581	9.44	17↓	76.8	70
41	M	3y	N	MPKU	D	E	312	150	7.2	22.6	9.9	–	30	669	21.1↑	38	108	44↓
42	F	19y 9m	N	MPKU	D	E	1521	882	7.5	42↑	10.2	–	13	387	21.6↑	33	95	73
43	M	3y	↑↑	MPKU	D	E	435	174	7	26.3	10.2	–	–	967	17.6↑	27	98	79
44	M	36y	N	CPKU	D	L	401	663	7.8	25.7	9.4	–	11↓	2000↑	28↑	29	71	84
45	M	4y 6m	N	CPKU	D	E	360	294	6.7	14.5↓	9.7	–	15	737	28↑	39	73	66
46	F	43y 1m	↑	CPKU	D	L	438	516	6.9	26.6	9.4	–	–	368	18.45↑	30	75	56↓
47	F	12y 1m	N	MPKU	D	E	587	132	6.9	20.2↓	9.6	–	–	290	13.72	28	68	73
48	F	8y 5m	N	MPKU	D	E	245	192	7.1	17.2↓	9.8	–	–	582	24↑	21	65	58↓
49	M	9y 4m	N	CPKU	D	E	552	264	7.2	29.1	9.9	–	23	616	24↑	25	85	64
50	M	44y 11m	N	CPKU	D	L	299	897	7.2	34	9.8	3.5	52	302	13.4	16.1↓	59↓	50↓
51	F	41y 8m	N	CPKU	D	L	765	751	7.5	29	9.6	2.6	15	482	43.9↑	20	73	70
52	M	39y 11m	↑	CPKU	D	L	600	816	7.6	26	10	3.1	40	698	28.9↑	27	77	58↓
53	F	39y 6m	N	CPKU	D	L	692	300	7.3	25	9.3	2.9	22	1767	32.5↑	27	74	54↓
54	F	38y 2m	↑↑	MPKU	D	L	478	766	7.1	24	10.1	3.2	42	401	24↑	22	84	67
55	F	34y 11m	N	CPKU	D	E	450	490	6.7	20↓	9.8	3.3	25	258	17.9↑	11↓	70	52↓
56	M	32y 2m	↑	CPKU	D	L	505	696	7.9	23	9.8	2.7	21	730	22.01↑	17↓	71	53↓
57	M	32y 1m	↑	CPKU	D	L	962	422	7.6	32	10.4	2.6	82	1031	49.6↑	28	98	122
58	F	31y 11m	N	CPKU	D	L	481	679	7.8	26	9.9	2.8	23	832	32.6↑	24	71	83
59	M	30y 7m	↑↑	CPKU	D	E	800	695	7.6	36	10.2	2.7	114	495	38.8↑	18↓	85	83
60	M	28y 6m	↑	MPKU	D/T	E	229	452	7.7	34	9.9	3.6	203	571	30.6↑	21.5	84	86
61	F	24y 11m	↑	CPKU	D	E	730	388	7.2	28	10	2.3↓	22	391	19.7↑	44↑	63↓	58↓
62	F	23y 8m	N	CPKU	D	E	237	431	7.5	28	9.8	2.6	18	1266	22.2↑	17↓	79	82
63	F	20y 11m	↑↑	CPKU	D	E	511	370	7.3	29	10.2	4.6	90	435	31.1↑	30	66	77
64	M	20y 9m	↑	CPKU	D	E	312	613	7.7	29	10.2	7.7↑	246	600	16.5	27	101	45↓
65	M	19y 4m	N	CPKU	D	E	700	567	6.4	20↓	10.4	3.2	–	337	7.9	33	104	–
66	F	19y 7m	N	MPKU	D/T	E	3051	262	7.2	37	9.9	4	32	584	20.6↑	31	67	76
67	F	19y 3m	↑↑	MPKU	D/T	E	461	197	7.7	28	10.3	3.7	14	419	10.4	17↓	75	87
68	F	18y 8m	↑	MPKU	D	E	2212	437	8	33	10.1	3.4	37	605	29.1↑	19.2↓	74	71
69	F	17y 4m	↑	MPKU	D/T	E	802	261	7.3	44↑	10.4	3.3	21	407	5.9	31	96	70
70	M	16y 8m	N	MPKU	D	E	1500	680	7.6	31	10.5	3	46	521	8.5	25	76	51↓
71	M	15y 4m	↓	MPKU	D/T	E	2585	231	7.3	20↓	10.2	4.8	40	589	16.7	21	74	69
72	F	15y 9m	N	MPKU	D/T	E	240	277	7.1	29	9.9	3.5	10↓	476	21.1↑	24	100	86
73	F	14y 10m	↑↑	CPKU	D	E	314	427	7.8	29	10.1	4	45	725	9.7	23	92	45↓
74	F	14y 8m	N	CPKU	D	E	2000	388	8.3	34	9.8	4.1	21	959	20.5↑	23	105	74
75	F	14y 5m	N	MPKU	D/T	E	513	199	8	27	9.8	3.3	17	583	12.9	18↓	75	71
76	M	13y 5m	N	CPKU	D	E	260	227	7.8	18↓	10.1	4	43	1189	11.1	33	83	74
77	F	12y 8m	↓	CPKU	D	E	399	363	7.7	25	10.1	4.5	39	911	31↑	28	86	71
78	M	10y 6m	↑	CPKU	D	E	261	430	7.1	23	10.3	3.8	29	1060	32.4↑	31	80	54↓
79	M	11y 5m	↑↑	CPKU	D	E	521	310	7.6	23	10.8	5	17	920	22.9↑	33	74	49↓
80	M	11y 4m	↑↑	MPKU	D	E	524	274	7.7	34	11↑	3.2	23	598	19.8↑	42	94	51↓
81	M	11y	N	CPKU	D	E	148	191	7	19↓	10.2	4.3	32	1192	16.2	27	94	84
82	F	9y 9m	↑↑	MPKU	D	E	1250	196	7.3	23	10.4	4.2	75	541	30.4↑	38	83	75
83	M	8y	N	MPKU	D/T	E	352	202	6.8	20↓	10.1	5.1	29	788	17.5	36	73	63
84	M	7y 10m	↑↑	CPKU	D	E	872	171	6.4	15↓	9.2	4.3	39	687	24↑	29	92	56↓
85	F	7y 8m	↑↑	MPKU	D/T	E	900	161	7.3	19↓	9.8	4.3	35	833	34.1↑	26	76	69
86	F	7y 8m	↑↑	MPKU	D/T	E	457	155	7.1	18↓	9.8	4.5	46	865	30.8↑	46↑	74	72
87	M	7y 2m	N	CPKU	D	E	815	160	7	23	9.5	4	28	1134	38.4↑	14↓	91	63
88	F	5y 7m	↑	MPKU	D/T	E	380	266	6.6	20↓	9.9	5.3	37	253	8.7	–	95	68
89	F	5y 6m	N	CPKU	D	E	1050	162	7.7	14↓	10	4.1	83	1293	32.5↑	25	91	86
90	F	4y	N	MPKU	D/T	E	264	195	6.9	20↓	10.4	5.7	6↓	748	11.5	26	66	75
91	M	3y 8m	N	MPKU	D	E	165	179	7	15↓	9.7	4.2	42	868	36.8↑	24	67	49↓
92	F	1y 10m	N	CPKU	D	E	174	93	7.1	13↓	10.1	5.3	27	947	26.9↑	22	98	59↓
93	M	1y 4m	N	CPKU	D	E	319	78	7	25	10.2	5.5	29	774	30.7↑	19↓	110	52↓
94	F	1y 4m	↑↑	CPKU	D	E	259	98	6.3	16↓	10.1	5.5	18	548	23.4↑	27	113	40↓
95	M	9m	↑	CPKU	D	E	222	222	6.8	14↓	10.7	5.6	25	1882	17.5	32	131	–
96	M	7m	N	CPKU	D	E	750	204	6.8	21	10.6	5.7	22	623	21↑	25	91	50↓
97	M	1y 11m	N	HPA	–	E	2601	250	6.9	18↓	10.2	4.9	23	290	10.1	18.7↓	73	73
98	F	5y 8m	N	HPA	–	E	3000	258	7.5	21	9.9	4.5	18	545	13.4	17↓	82	61
99	F	15y 2m	N	HPA	–	E	2700	280	8	23	9.8	3.6	6↓	730	–	29	74	76
100	F	6y 7m	↑	HPA	–	E	2600	240	7.1	27	9.6	5.1	22	542	10.8	17.3↓	68	95
101	F	9y	N	HPA	–	E	2400	270	7.4	20↓	10.1	4.8	57	474	18.8↑	30	67	67
102	F	8y	↑	HPA	–	E	1900	230	7.5	24	10.2	4.1	21	638	17.5	18↓	101	100
103	F	4y 8m	N	HPA	–	E	2800	225	6.8	20↓	9.6	4.7	24	738	13.6	31	88	93
104	M	4y	↑	HPA	–	E	600	180	6.9	22	9.7	5	12↓	547	18.6↑	21	110	54↓
105	M	1y 1m	N	HPA	–	E	2000	127	6.5	23	11.3↑	5.7	23	627	6.4	34	88	65
106	F	9y	N	HPA	–	E	1450	210	6.9	22	9.7	4.8	21	720	19↑	18↓	75	85
107	M	4y	↑↑	HPA	–	E	1350	300	7.3	16↓	9.9	5.1	9↓	488	4.1	27	73	82
108	F	3y 11m	↓	HPA	–	E	857	305	7.3	16↓	9.7	4.7	14	796	17.5	33.9	57↓	72
109	M	1y 1m	N	HPA	–	E	1500	320	6.6	20↓	9.5	5.1	41	727	23.8↑	17.6↓	101	66
110	F	7y 10m	N	HPA	–	E	722	230	7.2	15↓	10	4.3	43	1108	9	28	60↓	94
111	F	6y 3m	N	HPA	–	E	750	173	7.1	16↓	9.9	5.2	26	686	11.9	16.6↓	87	83
112	F	4y 1m	N	HPA	–	E	2000	175	6.5	16↓	9.5	4.6	42	905	19.1↑	33	72	90
113	F	3y 6m	N	HPA	–	E	2400	198	7.1	23	10.1	4.6	10↓	669	16.2	12↓	98	81
114	M	11y	N	HPA	–	E	2299	300	7.4	20↓	9.6	4.4	20	650	17.5	14↓	79	83
115	M	8y 1m	↑	HPA	–	E	1800	266	7	20↓	10.4	4.1	44	495	13.8	29	74	95
116	F	2y 10m	N	HPA	–	E	1900	97	7	20↓	10.4	5.3	27	483	17.5	30	65	106
117	F	5y 7m	N	HPA	–	E	1415	139	7	17↓	10.2	5.1	17	741	17	30.5	72	72
118	F	2y 11m	N	HPA	–	E	1450	283	6.3	21	9.3	3.9	94	435	17.5	30	62↓	61
119	F	3y 4m	N	HPA	–	E	2100	180	7.6	34	10.9↑	5.5	47	606	17.5	20	73	70
120	M	1y 6m	N	HPA	–	E	950	305	6.6	18↓	10.1	5.9	10↓	190	15.1	29	61↓	53↓
121	F	23y 9m	N	CPKU	D	E	235	1545	7.6	24	9.5	2.5↓	32	421	14	24	109	85
122	F	28y 1m	↑↑	CPKU	D	E	437	624	7.6	24	9.5	3.3	80	1289	17	41↑	65	69
123	M	22y 7m	N	CPKU	D	E	443	516	6.1↓	21	8.9	3.8	212	982	17	28	76	87
124	M	10y 4m	N	MPKU	D/T	E	922	220	7.7	26	11↑	4	42	435	12	27	71	86
125	F	17y 2m	N	CPKU	D	E	245	990	7.3	30	9.8	6.5↑	28	363	5.8	24	112	75
126	F	19y 19m	N	MPKU	D	E	312	882	7.5	35	10.2	2.2↓	41	387	17.5	33	95	73
127	M	8y 2m	↑↑	HPA	–	E	2100	290	7.5	21	10.1	4.6	23	790	10.4	42 ↑	70	93
128	M	13y 4m	N	CPKU	D	E	182	282	6.7	32	10.1	4.5	44	394	17.2	30	91	85
129	M	19y 11m	↑↑	CPKU	D	E	492	396	7.1	29	9.8	4.7	29	445	11.8	29	76	69
130	F	27y 9m	N	CPKU	D	E	365	516	6.6	24	8.7	3.4	40	1069	17.5	40	85	85
131	F	12y 7m	↑↑	MPKU	D/T	E	823	279	6.5	21	9.4	4.1	32	389	14	32	74	65
132	M	10y 4m	N	MPKU	D/T	E	922	222	7.5	26	9.8	3.8	55	437	12	27	70	86
133	M	18y 5m	↓	CPKU	D	E	256	195	6.9	34	9.8	4.3	17	269	10.2	28	76	74
134	F	42y 1m	↑	MPKU	D/T	E	855	756	7	29	9.6	4.3	–	366	14.7	35	116	68
135	F	11y 5m	↑↑	MPKU	D/T	E	2052	180	7.3	21	10.3	3.8	72	420	10.3	38	98	64
136	M	23y 5m	↑	CPKU	D	L	452	735	7.8	29	9.7	4.2	18	209	17	20	81	88
137	F	25y 4m	N	CPKU	D/T	E	730	803	7.4	31	9.9	2.8	42	229	4.7	27	69	89
138	F	21y11m	N	MPKU	D/T	E	3000	232	7.5	26	9.9	3.7	30	394	11.1	28	70	82
139	F	17y 10m	N	MPKU	D/T	E	802	436	7.5	29	9.5	3.4	22	571	17.5	22	98	72
140	F	14y	N	CPKU	D	E	413	204	7.6	32	10	4.1	43	727	11.1	31	86	68
141	M	13y	N	CPKU	D	E	260	449	8	37	10	4	50	905	17.5	33	101	85
142	F	10y 7m	↑↑	CPKU	D	E	148	476	7.5	24	9.9	4.1	29	605	13.7	27	113	67
143	M	10y 2m	N	MPKU	D/T	E	1600	245	6.8	25	10.2	4.3	39	699	16.1	27	68	87
144	F	3y 5m	↑↑	CPKU	D	E	264	169	6.6	22	10.4	5.2	27	1247	–	26	89	80
145	M	2y 8m	N	CPKU	D	E	165	76	6.6	24	10.5	4.2	25	1057	–	24	82	80
146	F	1y 5m	N	CPKU	D	E	174	248	7.2	24	10.4	4.9	20	877	–	23	95	65
147	F	5y 7m	N	HPA	–	E	1900	245	7.5	24	10.1	4.8	18	382	10.1	20	97	72
148	F	18y 10m	↑	HPA	–	E	2800	260	7.4	30	9.7	4	17	520	3.9	31	86	70
149	M	7y 11m	N	HPA	–	E	1450	235	7.5	21	10.8	4.5	21	730	17	20	77	75
150	M	11y 11m	N	HPA	–	E	2300	280	7.4	24	9.6	4.9	14	509	8.5	20	132	85
151	F	8y 6m	N	HPA	–	E	1169	270	6.8	21	9.5	4.5	48	356	41.5	20	90	69
152	M	1y 6m	N	HPA	–	E	950	139	6.8	22	9.7	4	30	342	9.6	29	78	70
153	M	3y 3m	N	HPA	–	E	1642	192	6.6	22	9.4	5.2	19	537	26	24	74	64
154	F	23y 2m	N	CPKU	D	L	451	1272	7.4	35	10.8	3.6	25	362	4.2	23	81	91
155	F	37y 3m	↑	CPKU	D	L	299	576	7.2	25	10.3	3.3	23	228	–	20	85	67
156	M	16y 5m	N	HPA	–	E	330	2000	7.1	22	10.1	3.4	22	640	15	33	74	87

Characteristics of Hyperphenylalaninaemia patients with altered values of the biochemical parameters studied. P: patient; M: male; F: female; BMI: body mass index; N: normal; ↑overweight, ↑↑ obesity, CPKU: classic phenylketonuria; MPKU: mild-moderate PKU; MHPA: moderate hyperphenylalaninaemia; Tre:Treatment; D: Dietetic; T: BH4 therapy; TD: time of diagnosis; E: Early; L:late; Phe tol: Phenylalanine tolerance; Prot: Total protein total; Prealb: Prealbumin; Fer: Ferritine.
