# Supplementary material for: Micronutrient in hyperphenylalaninemia
Source: Data Brief. 2015 Aug 1;4:614–21. doi: 10.1016/j.dib.2015.07.026 (PMC4543207; doi:10.1016/j.dib.2015.07.026)
Supplement: Supplementary file 1 — Supplementary material [file mmc1.doc]

| P | Sex | Age | BMI | Diag | Tre. D/T | TD | Phe tol mg/d | Phe median µM | Prot g/dL | Prealb mg/dL | Ca mg/dL | P mg/dL | Fer ng/mL | B12 pg/mL | Folic ng/mL | 25OHD ng/mL | Zn µg/dL | Se µg/dL |
| --- | --- | --- | --- | --- | --- | --- | --- | --- | --- | --- | --- | --- | --- | --- | --- | --- | --- | --- |
| 1 | F | 2y 11m | N | CPKU | D | E | 345 | 138 | 6,6 | 18↓ | 9,9 | 5,6 | 135 | 853 | 24↑ | 40 | 99 | 47↓ |
| 2 | M | 19y 3m | ↑ | CPKU | D | L | 217 | 380 | 7,9 | 25 | 9,8 | 3,3 | 36 | 823 | 24↑ | 38,7 | 117 | 74 |
| 3 | M | 2y 7m | N | CPKU | D | E | 223 | 258 | 7 | 17↓ | 10 | 5,6 | 29 | 789 | 15,1 | 40,2 | 76,5 | 106 |
| 4 | F | 2y 7m | ↑↑ | CPKU | D | E | 218 | 123 | 6,4 | 14↓ | 10,2 | 5,6 | 30 | 444 | 13 | 26 | 56,6↓ | 38,6↓ |
| 5 | M | 18y 2m | N | CPKU | D | E | 469 | 408 | 7,5 | 30 | 10,1 | 3,9 | 37 | 723 | 24↑ | 37,4 | 78,5 | 32,3↓ |
| 6 | M | 28y 9m | ↑ | CPKU | D | E | 367 | 1032 | 7,5 | 36 | 10,2 | 4 | 32 | 272 | 8,6 | 25,5 | 119 | 52,7↓ |
| 7 | F | 1y 2m | ↑ | CPKU | D | E | 118 | 162 | 6,5 | 14↓ | 9,2 | 4 | 84 | 696 | 18,7↑ | 36,2 | 81,5 | 68,7 |
| 8 | F | 15y 2m | N | CPKU | D | E | 454 | 480 | 7,7 | 28 | 10,4 | 4 | 126 | 405 | 17,3 | 19,1↓ | 70 | 70 |
| 9 | F | 2y 4m | ↓ | CPKU | D | E | 243 | 90 | 6,7 | 16↓ | 10,1 | 5,2 | 28 | 934 | 18,5↑ | 40 | 78,5 | 62,5 |
| 10 | F | 13y 1m | ↑↑ | CPKU | D | E | 390 | 474 | 6,8 | 22 | 9,6 | 5,2 | 5↓ | 347 | 24↑ | 34,4 | 82,5 | 69,5 |
| 11 | F | 3y 3m | N | CPKU | D | E | 289 | 225 | 7 | 14↓ | 10,3 | 4,7 | 40 | 835 | 13,2 | 29,3 | 59,7↓ | 19,6↓ |
| 12 | M | 4y 10m | N | CPKU | D | E | 379 | 207 | 6,8 | 17↓ | 10 | 5,1 | 28 | 462 | 17,2 | 27,8 | 106 | 62 |
| 13 | F | 92y 7m | ↑ | CPKU | D | E | 440 | 522 | 6,6 | 34 | 9,3 | 3,9 | 40 | 980 | 20↑ | 41,3 | 145↑ | 69,5 |
| 14 | F | 9y 6m | N | CPKU | D | E | 153 | 300 | 7,5 | 19↓ | 10,1 | 4,9 | 52 | 617 | 16,9 | 26,4 | 71,5 | 57↓ |
| 15 | M | 7y | N | CPKU | D/T | E | 1162 | 336 | 6,7 | 21 | 10,4 | 4,5 | 34 | 779 | 18,6↑ | 48↑ | 76,5 | 68,7 |
| 16 | M | 2y 6m | ↓ | CPKU | D | E | 473 | 348 | 6,4 | 22 | 9,8 | 5 | 21 | 468 | 24↑ | 40,6 | 76,5 | 58,7↓ |
| 17 | F | 15y 6m | N | CPKU | D | E | 447 | 450 | 7,7 | 19↓ | 10 | 4,6 | 32 | 388 | 5,68 | 27,2 | 101 | 71 |
| 18 | M | 39y | ↑ | CPKU | D | E | 205 | 777 | 6,5 | 31 | 9,4 | 2,3↓ | 89 | 354 | 16,5 | 27,6 | 113 | 51,7↓ |
| 19 | M | 5y 8m | ↑↑ | CPKU | D | E | 242 | 330 | 6,9 | 30 | 9,3 | 4,5 | 40 | 761 | 12 | 24 | 80 | 36,1↓ |
| 20 | M | 3y 2m | ↑↑ | CPKU | D | E | 194 | 204 | 6,8 | 21 | 10,1 | 9,9↑ | 24 | 560 | - | 34,2 | 78,5 | - |
| 21 | F | 14y 9m | ↑ | CPKU | D | E | 432 | 366 | 6,9 | 18↓ | 10,1 | 3,8 | 48 | 421 | 16,7 | 28,7 | 65 | 57↓ |
| 22 | M | 2y 9m | N | CPKU | D | E | 448 | 243 | 6,6 | 20↓ | 10,1 | 4,8 | 32 | 656 | 20,2↑ | 31,9 | 78,5 | 34↓ |
| 23 | M | 18y 10m | N | CPKU | D | L | 503 | 252 | 7,6 | 26 | 9,9 | 4,8 | 9,9↓ | 824 | 16,9 | 36,9 | 103 | 68,7 |
| 24 | F | 23y 11m | ↑ | CPKU | D | E | 320 | 381 | 7,4 | 28 | 9,3 | 3,5 | 61 | 628 | 16,3 | 41,3 | 144↑ | 68,7 |
| 25 | M | 3y 9m | ↑ | CPKU | D | E | 381 | 444 | 6,1↓ | 19↓ | 9,8 | 5,9 | 97 | 838 | 24↑ | 32,2 | 125 | 68,7 |
| 26 | F | 34y 9m | ↑↑ | CPKU | D | E | 409 | 1158 | 7,7 | 21 | 9,6 | 3 | 39 | 524 | 6,4 | 30,4 | 120 | 40,18↓ |
| 27 | M | 5y 10m | ↑ | CPKU | D | E | 425 | 258 | 7,2 | 16↓ | 9,6 | 5 | 13↓ | 675 | 15,1 | 19↓ | 73,5 | 74,9 |
| 28 | F | 30y 4m | N | CPKU | D | E | 771 | 972 | 6,4 | 21 | 8,5↓ | 2,7 | 22 | 301 | 11,5 | 20,2 | 75,5 | - |
| 29 | M | 3y 7m | N | CPKU | D | E | 226 | 192 | 6,3 | 16↓ | 10,7 | 5,9 | 8↓ | 679 | 16,3 | 36,5 | 80,5 | 68,7 |
| 30 | M | 23y | ↑ | CPKU | D | E | 256 | 846 | 7,2 | 37 | 10,1 | 2,3↓ | 91 | 471 | 14,4 | 41,9 | 145↑ | 77,2 |
| 31 | M | 9m | N | CPKU | D | E | 250 | 90 | 6,4 | 20↓ | 10,3 | 6,4↑ | 267 | 1201 | 17,2 | 37,5 | 170↑ | 77,2 |
| 32 | F | 17y 4m | ↑↑ | CPKU | D | L | 169 | 576 | 6,8 | 22 | 9,8 | 3,3 | 23 | 937 | 24↑ | 20 | 75,5 | 49,4↓ |
| 33 | M | 10y 6m | N | CPKU | D | E | 55 | 156 | 6,4 | 17↓ | 9,8 | 3,3 | 127 | 423 | 16 | 31,8 | 93 | 71 |
| 34 | F | 6y 4m | N | CPKU | D | E | 537 | 156 | 7,3 | 20↓ | 10,4 | 5,5 | 42 | 600 | 14,3 | 42 | 71,5 | 135↑ |
| 35 | M | 5y 11m | ↑ | CPKU | D | E | 403 | 138 | 6,5 | 18↓ | 9,9 | 5,1 | 24 | 372 | 16,9 | 52↑ | 74,5 | 124 |
| 36 | M | 5y 1m | N | CPKU | D | E | 489 | 384 | 6,7 | 19↓ | 10 | 4,5 | 27 | 1875 | 18,5↑ | 43,2↑ | 77 | 54,8↓ |
| 37 | M | 42y 6m | ↑ | CPKU | D | L | 121 | 888 | 7,6 | 24 | 9,2 | - | 73 | 660 | 30↑ | 26 | 69,2 | 60 |
| 38 | F | 5y 5m | ↑↑ | MPKU | D/T | E | 1069 | 198 | 6,7 | 18,4↓ | 10 | - | 35 | 501 | 30↑ | 33 | 87,3 | 42↓ |
| 39 | F | 4y 1m | N | MPKU | D | E | 438 | 117 | 7 | 22,8 | 10,2 | - | 34 | 766 | 28↑ | 36 | 67,2 | 48↓ |
| 40 | M | 26y 4m | ↑ | MPKU | D | E | 430 | 300 | 7 | 19,1↓ | 9 | - | 108 | 581 | 9,44 | 17↓ | 76,8 | 70 |
| 41 | M | 3y | N | MPKU | D | E | 312 | 150 | 7,2 | 22,6 | 9,9 | - | 30 | 669 | 21,1↑ | 38 | 108 | 44↓ |
| 42 | F | 19y 9m | N | MPKU | D | E | 1521 | 882 | 7,5 | 42↑ | 10,2 | - | 13 | 387 | 21,6↑ | 33 | 95 | 73 |
| 43 | M | 3y | ↑↑ | MPKU | D | E | 435 | 174 | 7 | 26,3 | 10,2 | - | - | 967 | 17,6↑ | 27 | 98 | 79 |
| 44 | M | 36y | N | CPKU | D | L | 401 | 663 | 7,8 | 25,7 | 9,4 | - | 11↓ | 2000↑ | 28↑ | 29 | 71 | 84 |
| 45 | M | 4y 6m | N | CPKU | D | E | 360 | 294 | 6,7 | 14,5↓ | 9,7 | - | 15 | 737 | 28↑ | 39 | 73 | 66 |
| 46 | F | 43y 1m | ↑ | CPKU | D | L | 438 | 516 | 6,9 | 26,6 | 9,4 | - | - | 368 | 18,45↑ | 30 | 75 | 56↓ |
| 47 | F | 12y 1m | N | MPKU | D | E | 587 | 132 | 6,9 | 20,2↓ | 9,6 | - | - | 290 | 13,72 | 28 | 68 | 73 |
| 48 | F | 8y 5m | N | MPKU | D | E | 245 | 192 | 7,1 | 17,2↓ | 9,8 | - | - | 582 | 24↑ | 21 | 65 | 58↓ |
| 49 | M | 9y 4m | N | CPKU | D | E | 552 | 264 | 7,2 | 29,1 | 9,9 | - | 23 | 616 | 24↑ | 25 | 85 | 64 |
| 50 | M | 44y 11m | N | CPKU | D | L | 299 | 897 | 7,2 | 34 | 9,8 | 3,5 | 52 | 302 | 13,4 | 16,1↓ | 59↓ | 50↓ |
| 51 | F | 41y 8m | N | CPKU | D | L | 765 | 751 | 7,5 | 29 | 9,6 | 2,6 | 15 | 482 | 43,9↑ | 20 | 73 | 70 |
| 52 | M | 39y 11m | ↑ | CPKU | D | L | 600 | 816 | 7,6 | 26 | 10 | 3,1 | 40 | 698 | 28,9↑ | 27 | 77 | 58↓ |
| 53 | F | 39y 6m | N | CPKU | D | L | 692 | 300 | 7,3 | 25 | 9,3 | 2,9 | 22 | 1767 | 32,5↑ | 27 | 74 | 54↓ |
| 54 | F | 38y 2m | ↑↑ | MPKU | D | L | 478 | 766 | 7,1 | 24 | 10,1 | 3,2 | 42 | 401 | 24↑ | 22 | 84 | 67 |
| 55 | F | 34y 11m | N | CPKU | D | E | 450 | 490 | 6,7 | 20↓ | 9,8 | 3,3 | 25 | 258 | 17,9↑ | 11↓ | 70 | 52↓ |
| 56 | M | 32y 2m | ↑ | CPKU | D | L | 505 | 696 | 7,9 | 23 | 9,8 | 2,7 | 21 | 730 | 22,01↑ | 17↓ | 71 | 53↓ |
| 57 | M | 32y 1m | ↑ | CPKU | D | L | 962 | 422 | 7,6 | 32 | 10,4 | 2,6 | 82 | 1031 | 49,6↑ | 28 | 98 | 122 |
| 58 | F | 31y 11m | N | CPKU | D | L | 481 | 679 | 7,8 | 26 | 9,9 | 2,8 | 23 | 832 | 32,6↑ | 24 | 71 | 83 |
| 59 | M | 30y 7m | ↑↑ | CPKU | D | E | 800 | 695 | 7,6 | 36 | 10,2 | 2,7 | 114 | 495 | 38,8↑ | 18↓ | 85 | 83 |
| 60 | M | 28y 6m | ↑ | MPKU | D/T | E | 229 | 452 | 7,7 | 34 | 9,9 | 3,6 | 203 | 571 | 30,6↑ | 21,5 | 84 | 86 |
| 61 | F | 24y 11m | ↑ | CPKU | D | E | 730 | 388 | 7,2 | 28 | 10 | 2,3↓ | 22 | 391 | 19,7↑ | 44↑ | 63↓ | 58↓ |
| 62 | F | 23y 8m | N | CPKU | D | E | 237 | 431 | 7,5 | 28 | 9,8 | 2,6 | 18 | 1266 | 22,2↑ | 17↓ | 79 | 82 |
| 63 | F | 20y 11m | ↑↑ | CPKU | D | E | 511 | 370 | 7,3 | 29 | 10,2 | 4,6 | 90 | 435 | 31,1↑ | 30 | 66 | 77 |
| 64 | M | 20y 9m | ↑ | CPKU | D | E | 312 | 613 | 7,7 | 29 | 10,2 | 7,7↑ | 246 | 600 | 16,5 | 27 | 101 | 45↓ |
| 65 | M | 19y 4m | N | CPKU | D | E | 700 | 567 | 6,4 | 20↓ | 10,4 | 3,2 | - | 337 | 7,9 | 33 | 104 | - |
| 66 | F | 19y 7m | N | MPKU | D/T | E | 3051 | 262 | 7,2 | 37 | 9,9 | 4 | 32 | 584 | 20,6↑ | 31 | 67 | 76 |
| 67 | F | 19y 3m | ↑↑ | MPKU | D/T | E | 461 | 197 | 7,7 | 28 | 10,3 | 3,7 | 14 | 419 | 10,4 | 17↓ | 75 | 87 |
| 68 | F | 18y 8m | ↑ | MPKU | D | E | 2212 | 437 | 8 | 33 | 10,1 | 3,4 | 37 | 605 | 29,1↑ | 19,2↓ | 74 | 71 |
| 69 | F | 17y 4m | ↑ | MPKU | D/T | E | 802 | 261 | 7,3 | 44↑ | 10,4 | 3,3 | 21 | 407 | 5,9 | 31 | 96 | 70 |
| 70 | M | 16y 8m | N | MPKU | D | E | 1500 | 680 | 7,6 | 31 | 10,5 | 3 | 46 | 521 | 8,5 | 25 | 76 | 51↓ |
| 71 | M | 15y 4m | ↓ | MPKU | D/T | E | 2585 | 231 | 7,3 | 20↓ | 10,2 | 4,8 | 40 | 589 | 16,7 | 21 | 74 | 69 |
| 72 | F | 15y 9m | N | MPKU | D/T | E | 240 | 277 | 7,1 | 29 | 9,9 | 3,5 | 10↓ | 476 | 21,1↑ | 24 | 100 | 86 |
| 73 | F | 14y 10m | ↑↑ | CPKU | D | E | 314 | 427 | 7,8 | 29 | 10,1 | 4 | 45 | 725 | 9,7 | 23 | 92 | 45↓ |
| 74 | F | 14y 8m | N | CPKU | D | E | 2000 | 388 | 8,3 | 34 | 9,8 | 4,1 | 21 | 959 | 20,5↑ | 23 | 105 | 74 |
| 75 | F | 14y 5m | N | MPKU | D/T | E | 513 | 199 | 8 | 27 | 9,8 | 3,3 | 17 | 583 | 12,9 | 18↓ | 75 | 71 |
| 76 | M | 13y 5m | N | CPKU | D | E | 260 | 227 | 7,8 | 18↓ | 10,1 | 4 | 43 | 1189 | 11,1 | 33 | 83 | 74 |
| 77 | F | 12y 8m | ↓ | CPKU | D | E | 399 | 363 | 7,7 | 25 | 10,1 | 4,5 | 39 | 911 | 31↑ | 28 | 86 | 71 |
| 78 | M | 10y 6m | ↑ | CPKU | D | E | 261 | 430 | 7,1 | 23 | 10,3 | 3,8 | 29 | 1060 | 32,4↑ | 31 | 80 | 54↓ |
| 79 | M | 11y 5m | ↑↑ | CPKU | D | E | 521 | 310 | 7,6 | 23 | 10,8 | 5 | 17 | 920 | 22,9↑ | 33 | 74 | 49↓ |
| 80 | M | 11y 4m | ↑↑ | MPKU | D | E | 524 | 274 | 7,7 | 34 | 11↑ | 3,2 | 23 | 598 | 19,8↑ | 42 | 94 | 51↓ |
| 81 | M | 11y | N | CPKU | D | E | 148 | 191 | 7 | 19↓ | 10,2 | 4,3 | 32 | 1192 | 16,2 | 27 | 94 | 84 |
| 82 | F | 9y 9m | ↑↑ | MPKU | D | E | 1250 | 196 | 7,3 | 23 | 10,4 | 4,2 | 75 | 541 | 30,4↑ | 38 | 83 | 75 |
| 83 | M | 8y | N | MPKU | D/T | E | 352 | 202 | 6,8 | 20↓ | 10,1 | 5,1 | 29 | 788 | 17,5 | 36 | 73 | 63 |
| 84 | M | 7y 10m | ↑↑ | CPKU | D | E | 872 | 171 | 6,4 | 15↓ | 9,2 | 4,3 | 39 | 687 | 24↑ | 29 | 92 | 56↓ |
| 85 | F | 7y 8m | ↑↑ | MPKU | D/T | E | 900 | 161 | 7,3 | 19↓ | 9,8 | 4,3 | 35 | 833 | 34,1↑ | 26 | 76 | 69 |
| 86 | F | 7y 8m | ↑↑ | MPKU | D/T | E | 457 | 155 | 7,1 | 18↓ | 9,8 | 4,5 | 46 | 865 | 30,8↑ | 46↑ | 74 | 72 |
| 87 | M | 7y 2m | N | CPKU | D | E | 815 | 160 | 7 | 23 | 9,5 | 4 | 28 | 1134 | 38,4↑ | 14↓ | 91 | 63 |
| 88 | F | 5y 7m | ↑ | MPKU | D/T | E | 380 | 266 | 6,6 | 20↓ | 9,9 | 5,3 | 37 | 253 | 8,7 | - | 95 | 68 |
| 89 | F | 5y 6m | N | CPKU | D | E | 1050 | 162 | 7,7 | 14↓ | 10 | 4,1 | 83 | 1293 | 32,5↑ | 25 | 91 | 86 |
| 90 | F | 4y | N | MPKU | D/T | E | 264 | 195 | 6,9 | 20↓ | 10,4 | 5,7 | 6↓ | 748 | 11,5 | 26 | 66 | 75 |
| 91 | M | 3y 8m | N | MPKU | D | E | 165 | 179 | 7 | 15↓ | 9,7 | 4,2 | 42 | 868 | 36,8↑ | 24 | 67 | 49↓ |
| 92 | F | 1y 10m | N | CPKU | D | E | 174 | 93 | 7,1 | 13↓ | 10,1 | 5,3 | 27 | 947 | 26,9↑ | 22 | 98 | 59↓ |
| 93 | M | 1y 4m | N | CPKU | D | E | 319 | 78 | 7 | 25 | 10,2 | 5,5 | 29 | 774 | 30,7↑ | 19↓ | 110 | 52↓ |
| 94 | F | 1y 4m | ↑↑ | CPKU | D | E | 259 | 98 | 6,3 | 16↓ | 10,1 | 5,5 | 18 | 548 | 23,4↑ | 27 | 113 | 40↓ |
| 95 | M | 9m | ↑ | CPKU | D | E | 222 | 222 | 6,8 | 14↓ | 10,7 | 5,6 | 25 | 1882 | 17,5 | 32 | 131 | - |
| 96 | M | 7m | N | CPKU | D | E | 750 | 204 | 6,8 | 21 | 10,6 | 5,7 | 22 | 623 | 21↑ | 25 | 91 | 50↓ |
| 97 | M | 1y 11m | N | HPA | - | E | 2601 | 250 | 6,9 | 18↓ | 10,2 | 4,9 | 23 | 290 | 10,1 | 18,7↓ | 73 | 73 |
| 98 | F | 5y 8m | N | HPA | - | E | 3000 | 258 | 7,5 | 21 | 9,9 | 4,5 | 18 | 545 | 13,4 | 17↓ | 82 | 61 |
| 99 | F | 15y 2m | N | HPA | - | E | 2700 | 280 | 8 | 23 | 9,8 | 3,6 | 6↓ | 730 | - | 29 | 74 | 76 |
| 100 | F | 6y 7m | ↑ | HPA | - | E | 2600 | 240 | 7,1 | 27 | 9,6 | 5,1 | 22 | 542 | 10,8 | 17,3↓ | 68 | 95 |
| 101 | F | 9y | N | HPA | - | E | 2400 | 270 | 7,4 | 20↓ | 10,1 | 4,8 | 57 | 474 | 18,8↑ | 30 | 67 | 67 |
| 102 | F | 8y | ↑ | HPA | - | E | 1900 | 230 | 7,5 | 24 | 10,2 | 4,1 | 21 | 638 | 17,5 | 18↓ | 101 | 100 |
| 103 | F | 4y 8m | N | HPA | - | E | 2800 | 225 | 6,8 | 20↓ | 9,6 | 4,7 | 24 | 738 | 13,6 | 31 | 88 | 93 |
| 104 | M | 4y | ↑ | HPA | - | E | 600 | 180 | 6,9 | 22 | 9,7 | 5 | 12↓ | 547 | 18,6↑ | 21 | 110 | 54↓ |
| 105 | M | 1y 1m | N | HPA | - | E | 2000 | 127 | 6,5 | 23 | 11,3↑ | 5,7 | 23 | 627 | 6,4 | 34 | 88 | 65 |
| 106 | F | 9y | N | HPA | - | E | 1450 | 210 | 6,9 | 22 | 9,7 | 4,8 | 21 | 720 | 19↑ | 18↓ | 75 | 85 |
| 107 | M | 4y | ↑↑ | HPA | - | E | 1350 | 300 | 7,3 | 16↓ | 9,9 | 5,1 | 9↓ | 488 | 4,1 | 27 | 73 | 82 |
| 108 | F | 3y 11m | ↓ | HPA | - | E | 857 | 305 | 7,3 | 16↓ | 9,7 | 4,7 | 14 | 796 | 17,5 | 33,9 | 57↓ | 72 |
| 109 | M | 1y 1m | N | HPA | - | E | 1500 | 320 | 6,6 | 20↓ | 9,5 | 5,1 | 41 | 727 | 23,8↑ | 17,6↓ | 101 | 66 |
| 110 | F | 7y 10m | N | HPA | - | E | 722 | 230 | 7,2 | 15↓ | 10 | 4,3 | 43 | 1108 | 9 | 28 | 60↓ | 94 |
| 111 | F | 6y 3m | N | HPA | - | E | 750 | 173 | 7,1 | 16↓ | 9,9 | 5,2 | 26 | 686 | 11,9 | 16,6↓ | 87 | 83 |
| 112 | F | 4y 1m | N | HPA | - | E | 2000 | 175 | 6,5 | 16↓ | 9,5 | 4,6 | 42 | 905 | 19,1↑ | 33 | 72 | 90 |
| 113 | F | 3y 6m | N | HPA | - | E | 2400 | 198 | 7,1 | 23 | 10,1 | 4,6 | 10↓ | 669 | 16,2 | 12↓ | 98 | 81 |
| 114 | M | 11y | N | HPA | - | E | 2299 | 300 | 7,4 | 20↓ | 9,6 | 4,4 | 20 | 650 | 17,5 | 14↓ | 79 | 83 |
| 115 | M | 8y 1m | ↑ | HPA | - | E | 1800 | 266 | 7 | 20↓ | 10,4 | 4,1 | 44 | 495 | 13,8 | 29 | 74 | 95 |
| 116 | F | 2y 10m | N | HPA | - | E | 1900 | 97 | 7 | 20↓ | 10,4 | 5,3 | 27 | 483 | 17,5 | 30 | 65 | 106 |
| 117 | F | 5y 7m | N | HPA | - | E | 1415 | 139 | 7 | 17↓ | 10,2 | 5,1 | 17 | 741 | 17 | 30,5 | 72 | 72 |
| 118 | F | 2y 11m | N | HPA | - | E | 1450 | 283 | 6,3 | 21 | 9,3 | 3,9 | 94 | 435 | 17,5 | 30 | 62↓ | 61 |
| 119 | F | 3y 4m | N | HPA | - | E | 2100 | 180 | 7,6 | 34 | 10,9↑ | 5,5 | 47 | 606 | 17,5 | 20 | 73 | 70 |
| 120 | M | 1y 6m | N | HPA | - | E | 950 | 305 | 6,6 | 18↓ | 10,1 | 5,9 | 10↓ | 190 | 15,1 | 29 | 61↓ | 53↓ |
| 121 | F | 23y9m | N | CPKU | D | E | 235 | 1545 | 7,6 | 24 | 9,5 | 2,5↓ | 32 | 421 | 14 | 24 | 109 | 85 |
| 122 | F | 28y1m | ↑↑ | CPKU | D | E | 437 | 624 | 7,6 | 24 | 9,5 | 3,3 | 80 | 1289 | 17 | 41↑ | 65 | 69 |
| 123 | M | 22y7m | N | CPKU | D | E | 443 | 516 | 6,1↓ | 21 | 8,9 | 3,8 | 212 | 982 | 17 | 28 | 76 | 87 |
| 124 | M | 10y4m | N | MPKU | D/T | E | 922 | 220 | 7,7 | 26 | 11↑ | 4 | 42 | 435 | 12 | 27 | 71 | 86 |
| 125 | F | 17y2m | N | CPKU | D | E | 245 | 990 | 7,3 | 30 | 9,8 | 6,5↑ | 28 | 363 | 5,8 | 24 | 112 | 75 |
| 126 | F | 19y19m | N | MPKU | D | E | 312 | 882 | 7,5 | 35 | 10,2 | 2,2↓ | 41 | 387 | 17,5 | 33 | 95 | 73 |
| 127 | M | 8y2m | ↑↑ | HPA | - | E | 2100 | 290 | 7,5 | 21 | 10,1 | 4,6 | 23 | 790 | 10,4 | 42 ↑ | 70 | 93 |
| 128 | M | 13y4m | N | CPKU | D | E | 182 | 282 | 6,7 | 32 | 10,1 | 4,5 | 44 | 394 | 17,2 | 30 | 91 | 85 |
| 129 | M | 19y11m | ↑↑ | CPKU | D | E | 492 | 396 | 7,1 | 29 | 9,8 | 4,7 | 29 | 445 | 11,8 | 29 | 76 | 69 |
| 130 | F | 27y9m | N | CPKU | D | E | 365 | 516 | 6,6 | 24 | 8,7 | 3,4 | 40 | 1069 | 17,5 | 40 | 85 | 85 |
| 131 | F | 12y7m | ↑↑ | MPKU | D/T | E | 823 | 279 | 6,5 | 21 | 9,4 | 4,1 | 32 | 389 | 14 | 32 | 74 | 65 |
| 132 | M | 10y4m | N | MPKU | D/T | E | 922 | 222 | 7,5 | 26 | 9,8 | 3,8 | 55 | 437 | 12 | 27 | 70 | 86 |
| 133 | M | 18y5m | ↓ | CPKU | D | E | 256 | 195 | 6,9 | 34 | 9,8 | 4,3 | 17 | 269 | 10,2 | 28 | 76 | 74 |
| 134 | F | 42y1m | ↑ | MPKU | D/T | E | 855 | 756 | 7 | 29 | 9,6 | 4,3 | - | 366 | 14,7 | 35 | 116 | 68 |
| 135 | F | 11y5m | ↑↑ | MPKU | D/T | E | 2052 | 180 | 7,3 | 21 | 10,3 | 3,8 | 72 | 420 | 10,3 | 38 | 98 | 64 |
| 136 | M | 23y5m | ↑ | CPKU | D | L | 452 | 735 | 7,8 | 29 | 9,7 | 4,2 | 18 | 209 | 17 | 20 | 81 | 88 |
| 137 | F | 25y4m | N | CPKU | D/T | E | 730 | 803 | 7,4 | 31 | 9,9 | 2,8 | 42 | 229 | 4,7 | 27 | 69 | 89 |
| 138 | F | 21y11m | N | MPKU | D/T | E | 3000 | 232 | 7,5 | 26 | 9,9 | 3,7 | 30 | 394 | 11,1 | 28 | 70 | 82 |
| 139 | F | 17y10m | N | MPKU | D/T | E | 802 | 436 | 7,5 | 29 | 9,5 | 3,4 | 22 | 571 | 17,5 | 22 | 98 | 72 |
| 140 | F | 14y | N | CPKU | D | E | 413 | 204 | 7,6 | 32 | 10 | 4,1 | 43 | 727 | 11,1 | 31 | 86 | 68 |
| 141 | M | 13y | N | CPKU | D | E | 260 | 449 | 8 | 37 | 10 | 4 | 50 | 905 | 17,5 | 33 | 101 | 85 |
| 142 | F | 10y7m | ↑↑ | CPKU | D | E | 148 | 476 | 7,5 | 24 | 9,9 | 4,1 | 29 | 605 | 13,7 | 27 | 113 | 67 |
| 143 | M | 10y2m | N | MPKU | D/T | E | 1600 | 245 | 6,8 | 25 | 10,2 | 4,3 | 39 | 699 | 16,1 | 27 | 68 | 87 |
| 144 | F | 3y5m | ↑↑ | CPKU | D | E | 264 | 169 | 6,6 | 22 | 10,4 | 5,2 | 27 | 1247 | - | 26 | 89 | 80 |
| 145 | M | 2y8m | N | CPKU | D | E | 165 | 76 | 6,6 | 24 | 10,5 | 4,2 | 25 | 1057 | - | 24 | 82 | 80 |
| 146 | F | 1y5m | N | CPKU | D | E | 174 | 248 | 7,2 | 24 | 10,4 | 4,9 | 20 | 877 | - | 23 | 95 | 65 |
| 147 | F | 5y7m | N | HPA | - | E | 1900 | 245 | 7,5 | 24 | 10,1 | 4,8 | 18 | 382 | 10,1 | 20 | 97 | 72 |
| 148 | F | 18y10m | ↑ | HPA | - | E | 2800 | 260 | 7,4 | 30 | 9,7 | 4 | 17 | 520 | 3,9 | 31 | 86 | 70 |
| 149 | M | 7y11m | N | HPA | - | E | 1450 | 235 | 7,5 | 21 | 10,8 | 4,5 | 21 | 730 | 17 | 20 | 77 | 75 |
| 150 | M | 11y11m | N | HPA | - | E | 2300 | 280 | 7,4 | 24 | 9,6 | 4,9 | 14 | 509 | 8,5 | 20 | 132 | 85 |
| 151 | F | 8y6m | N | HPA | - | E | 1169 | 270 | 6,8 | 21 | 9,5 | 4,5 | 48 | 356 | 41,5 | 20 | 90 | 69 |
| 152 | M | 1y6m | N | HPA | - | E | 950 | 139 | 6,8 | 22 | 9,7 | 4 | 30 | 342 | 9,6 | 29 | 78 | 70 |
| 153 | M | 3y3m | N | HPA | - | E | 1642 | 192 | 6,6 | 22 | 9,4 | 5,2 | 19 | 537 | 26 | 24 | 74 | 64 |
| 154 | F | 23y2m | N | CPKU | D | L | 451 | 1272 | 7,4 | 35 | 10,8 | 3,6 | 25 | 362 | 4,2 | 23 | 81 | 91 |
| 155 | F | 37y3m | ↑ | CPKU | D | L | 299 | 576 | 7,2 | 25 | 10,3 | 3,3 | 23 | 228 | - | 20 | 85 | 67 |
| 156 | M | 16y5m | N | HPA | - | E | 330 | 2000 | 7,1 | 22 | 10,1 | 3,4 | 22 | 640 | 15 | 33 | 74 | 87 |

Characteristics of Hyperphenylalaninaemia patients with altered values of the biochemical parameters studied

P: patient; M: male; F: female; BMI: body mass index; N: normal; ↑overweight, ↑↑ obesity, CPKU: classic phenylketonuria; MPKU: mild-moderate PKU; MHPA: moderate hyperphenylalaninaemia; Tre:Treatment; D: Dietetic; T: BH4 therapy; TD: time of diagnosis; E: Early; L:late; Phe tol: Phenylalanine tolerance; Prot: Total protein total; Prealb: Prealbumin; Fer: Ferritine
